# Response to the Letter by Spurling and Colleagues

**DOI:** 10.1186/s13054-023-04352-6

**Published:** 2023-02-20

**Authors:** Craig I. Coleman, Olivia S. Costa, Stuart J. Connolly, Mukul Sharma, Jan Beyer-Westendorf, Mary J. Christoph-Schubel, Belinda Lovelace

**Affiliations:** 1 Department of Pharmacy Practice, University of Connecticut School of Pharmacy, 69 North Eagleville Road, Unit 3092, Storrs, CT 06269 USA; 2 Evidence-Based Practice Center, Hartford Hospital, Hartford, CT USA; 3 McMaster University, Hamilton, ON Canada; 4 Population Health Research Institute, Hamilton, ON Canada; 5 Thrombosis and Anticoagulation Service, Division Hematology and Hemostaseology, Department of Medicine I, Dresden University Hospital, Dresden, Germany; 6 Department of Global Medical and Payer Evidence, AstraZeneca Pharmaceuticals, South San Francisco, CA USA; 7 Department of Health Economics and Payer Evidence, AstraZeneca Pharmaceuticals, Gaithersburg, MD USA

We would like to respond to Spurling et al. in their Letter to the Editor [1].

Spurling et al. questioned the bleed severity in our study [2] given the exclusion of Glasgow Coma Scale (GCS) Score < 7. Understanding outcomes in the most severe hemorrhages is important but poses an ethical challenge in a clinical trial setting wherein patients may not be able to appropriately express consent, and was outside the scope of this study of providing a synthetic trial arm comparison to ANNEXA-4. Notably, GCS scores were similar between andexanet alfa and 4-factor prothrombin complex concentrate (4F-PCC) patients even prior to weighting, suggesting comparable severity.

The authors note most 4F-PCC patients received a 25 U/kg dose instead of the 50 U/kg dose suggested by some societies, concluding the low-dose 4F-PCC may not have provided adequate clotting factor supplementation to achieve hemostasis. While we acknowledge that some (not all) guidelines recommend 50 U/kg, this recommendation is based upon limited data and neither dose has been assessed in clinical trials and been proven safe or efficacious or is approved by authorities for this indication. Prior studies comparing low- and high-dose 4F-PCC for reversal of oral FXai have demonstrated 25 U/kg is used more frequently in routine practice, and neither hemostatic effectiveness nor in-hospital mortality appear to be superior with the 50 U/kg dose versus 25 U/kg [3, 4]. In vitro data have shown that even at high doses, 4F-PCCs may only be able to normalize thrombin generation over a narrow range of low FXa inhibitor (FXai) concentrations [5].

Spurling et al. also noted that our study did not assess timing of the last dose of oral factor Xa inhibitor or baseline anti–factor Xa (anti-FXa) activity in all patients receiving 4F-PCC. All andexanet alfa patients in our study were subjects of the ANNEXA-4 efficacy population and required to have baseline anti-FXa activity ≥ 75 ng per milliliter. Spurling et al. are correct that 4F-PCC patients, assumed to have had their last FXai dose within 24 h, could have been included in our comparator arm with low or no circulating anticoagulant plasma levels. However, any bias due to low anti-FXa activity in the 4F-PCC arm would therefore enhance favorable outcomes in the comparator cohort.

While our propensity score-weighted comparative study design is not a substitute for a head-to-head randomized comparison, until the completion of ANNEXA-I we maintain this indirect comparison provides additional information to support clinicians in making treatment decisions.

## Data Availability

Not applicable.
